# SARS-CoV-2 RNA Detection with Duplex-Specific Nuclease Signal Amplification

**DOI:** 10.3390/mi12020197

**Published:** 2021-02-14

**Authors:** Meiqing Liu, Haoran Li, Yanwei Jia, Pui-In Mak, Rui P. Martins

**Affiliations:** 1 State-Key Laboratory of Analog and Mixed-Signal VLSI, Institute of Microelectronics, University of Macau, Macau 999078, China; mqliu@um.edu.mo (M.L.); harryleelhr@outlook.com (H.L.); pimak@um.edu.mo (P.-I.M.); rmartins@um.edu.mo (R.P.M.); 2 Faculty of Science and Technology–ECE, University of Macau, Macau 999078, China; 3 Faculty of Health Sciences, University of Macau, Macau 999078, China; 4 On Leave from Instituto Superior Técnico, Universidade de Lisboa, 1049-001 Lisboa, Portugal

**Keywords:** SARS-CoV-2, DNA probe, duplex-specific nuclease, signal amplification, RNA detection

## Abstract

The emergence of the novel severe acute respiratory syndrome coronavirus 2 (SARS-CoV-2), a zoonotic pathogen, has led to the outbreak of coronavirus disease 2019 (COVID-19) pandemic and brought serious threats to public health worldwide. The gold standard method for SARS-CoV-2 detection requires both reverse transcription (RT) of the virus RNA to cDNA and then polymerase chain reaction (PCR) for the cDNA amplification, which involves multiple enzymes, multiple reactions and a complicated assay optimization process. Here, we developed a duplex-specific nuclease (DSN)-based signal amplification method for SARS-CoV-2 detection directly from the virus RNA utilizing two specific DNA probes. These specific DNA probes can hybridize to the target RNA at different locations in the nucleocapsid protein gene (N gene) of SARS-CoV-2 to form a DNA/RNA heteroduplex. DSN cleaves the DNA probe to release fluorescence, while leaving the RNA strand intact to be bound to another available probe molecule for further cleavage and fluorescent signal amplification. The optimized DSN amount, incubation temperature and incubation time were investigated in this work. Proof-of-principle SARS-CoV-2 detection was demonstrated with a detection sensitivity of 500 pM virus RNA. This simple, rapid, and direct RNA detection method is expected to provide a complementary method for the detection of viruses mutated at the PCR primer-binding regions for a more precise detection.

## 1. Introduction

The emergence of the novel severe acute respiratory syndrome coronavirus (SARS-CoV-2) causing the coronavirus disease 2019 (COVID-19) pandemic has brought serious threats to public health worldwide [1,2]. The human-to-human transmission, the animal-to-human transmission, the asymptomatic transmission, and a long incubation time have made the disease control of COVID-19 a challenge for all governments around the world [3,4]. Virus testing, especially a rapid and precise testing, has been the key factor for the control of the pandemic before an effective vaccine is used in populations. 

Many methods based on traditional molecular diagnostics have been developed for COVID-19 detection and more novel methods utilizing nanoparticles as the indicators are under development [5,6,7,8,9,10,11,12,13,14]. Those methods mainly fall into two categories, a serological test and nucleic acid test. A serological test is an immunoassay that detects the SARS-CoV-2-specific antibodies, IgM and IgG, present in a patient’s serum. As long as a patient has been infected with SARS-CoV-2, there will be traces of antibodies, IgM and IgG, in the blood. Therefore, a serological test can be used as a retroactive investigation of the pandemic. However, it typically takes 5 to 10 days for a patient to generate antibodies [15]. Even worse, it was observed in clinics that some patients generated no antibody or little antibody after infection, making early detection based on a serological test impossible. 

Alternatively, a nucleic acid test directly detects the RNA of SARS-CoV-2 in the sample from a patient’s throat or lung lavage fluid, which can better reflect virus infection and offer the earliest detection [8]. Since SARS-CoV-2 is an RNA virus, reverse transcription polymerase chain reaction (RT-PCR) is utilized for the virus detection [16]. RT-PCR is a two-step reaction including a reverse transcription (RT) step to convert RNA to cDNA and a subsequent polymerase chain reaction (PCR) step to amplify a segment of the cDNA, either in a single tube or in separate tubes [17,18]. The specific binding of the RT primer and the pair of PCR primers guarantee the high specificity, high sensitivity, and high efficiency of the amplification [19,20]. Currently, RT-PCR has been regarded as the gold standard method for the clinical diagnosis of COVID-19 [16]. 

However, SARS-CoV-2 is an RNA virus, which mutates rapidly from generation to generation. Once a mutation occurs in the sequence of either the RT primer or the PCR primer-binding regions, the primer would fail to bind and lead to a false negative result. Collective genetic data have demonstrated that changes in the viral genome due to nucleotide insertion, deletion, or recombination and interchange among viruses were frequently observed [21,22]. One study has demonstrated that RT-PCR only showed a positive test rate of 38% in a total of 4880 specimens with a significant number of false negative cases [23]. Although genome sequencing can give much more information on the virus mutations and development, it requires professional technicians and expensive equipment under stringent laboratory conditions. Therefore, its wide usage in fast and populational COVID-19 tests is quite limited [24,25,26,27,28]. Most critically, both RT-PCR and genomic sequencing require an RT step before amplification or sequencing. This additional step includes another RT enzyme, which requires specific reaction conditions that are different from PCR reactions. It raises the complexity of the assay and makes the assay optimization time-consuming. A rapid, simple, accurate, and direct RNA detection method is in high demand for early diagnosis and pandemic control. 

Duplex-specific nuclease (DSN) is an enzyme isolated from the hepatopancreas of the Kamchatka crab [29]. It displays a considerable preference for cleaving the DNA strand in the double-stranded DNA or DNA/RNA heteroduplexes and requires at least about 10 bp DNA or a DNA/RNA perfect duplex. At the same time, it is practically inactive toward single-stranded DNA, or single- or double-stranded RNA. Moreover, this enzyme possesses a good discrimination between perfectly and non-perfectly matched duplexes [30]. The DSN enzyme has been widely applied in nucleic acid analysis [31,32,33,34], such as full-length cDNA library normalization [35], genomic single nucleotide polymorphism detection [29], and microRNA detection [36]. However, its application in virus detection is seldom reported. 

**Scheme 1:**
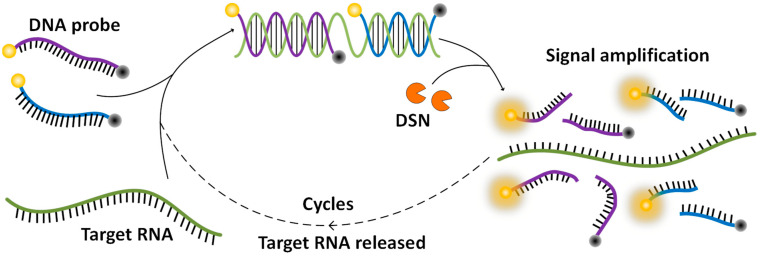
Schematics of duplex-specific nuclease signal amplification.

In this work, by taking advantage of the characteristics of DSN in cleaving the DNA strand but leaving the RNA strand untouched, we innovatively developed a DSN-based assay for a SARS-CoV-2 test. Scheme 1 shows the working principle of this method. DNA probes are specifically designed reverse complement to a segment of the RNA sequence in the nucleocapsid protein gene of SARS-CoV-2. A fluorophore is labeled at one end of the probe and a quencher is labeled at the other end to quench the fluorescence. The probes bind to the virus RNA to form DNA/RNA heteroduplexes when a virus is presented. DSN utilized in the assay cuts the DNA probe in the heteroduplex into pieces, which separates the fluorophore from the quencher and releases fluorescence. Then the RNA strand is free for another probe to bind, and then to be cut to release fluorescence. With the cycles going on, fluorescence builds up to a detectable level for an indication of the existence of the virus. Different from the current RT-PCR assay, this DSN-based assay directly amplifies from the virus RNA, making the assay simple to develop and implement. Furthermore, if a DNA probe were designed targeted to the recognition sequence of a primer, the mutated virus missed by an RT-PCR assay would be picked up with this DSN-based assay. Therefore, this DSN-based signal amplification method allows a rapid and simple detection of SARS-CoV-2, and can work as a complementary assay of RT-PCR to minimize the false negative results due to virus mutations.

## 2. Materials and Methods

### 2.1. Materials 

All synthetic DNA and RNA oligonucleotides (HPLC purified) were purchased from Sangon Biotechnology Co., Ltd. (Shanghai, China). The DNA strands were dissolved in ultrapure water and RNA strands were dissolved in diethylpyrocarbonate (DEPC) water for a stock concentration of 100 μM. The stock solutions were kept at −80 °C in aliquots of 20 µL. Lower concentrations of oligonucleotides were obtained by serial dilutions from the stock solutions. The sequences of the RNA and DNA oligonucleotides are listed in Table 1. Regions for DNA probe binding are underlined. The melting temperatures (T_m_) were estimated by the OligoAnalyzer Tool from Integrated DNA Technologies. Duplex-specific nuclease (DSN) was obtained from Evrogen Joint Stock Company (Moscow, Russia). All other chemicals were purchased from Sigma-Aldrich (Louis, MO, USA) and used without further purification. The tips and tubes purchased from Thermo Fisher (Waltham, MA., USA) were RNase free. No further treatment was performed for RNase inactivation. 

### 2.2. Methods

DSN stock solution was prepared by diluting 100 U lyophilized DSN enzyme in 100 µL DSN storage buffer (50 mM Tris-HCl, pH 8.0), and mixed by gently flicking the tube. Subsequently, the tube was spun briefly, and then incubated at room temperature for 5 min. Following that, 100 µL 100% glycerol was added to the tube to make a final concentration of 0.5 U/μL DSN with 50% glycerol. Various volumes of DSN stock solutions were added in a reaction to reach different DSN concentrations. 

DSN-based signal amplification was carried out in a 200 μL tube with a volume of 20 µL reaction mixture containing 1×DSN buffer (50 mM Tris-HCl, pH 8.0; 5 mM MgCl_2_, 1 mM DTT), enzyme (dissolved in 25 mM Tris-HCl, pH 8.0; 50% glycerol), DNA probe, and the target RNA. The concentrations of DSN, DNA probes, and the target RNA were tested at various concentrations for an optimized assay. The mixed solution was prepared at room temperature and then transferred to a C1000™ Thermal Cycler (Bio-Rad, Hercules, CA, USA) for incubation at various temperatures for 30–60 min. The specific concentrations of probes, targets, and DSN were adjusted in different experiments and are mentioned in the results section. After the reaction, the mixture was transferred to a 384-well microplate and scanned by a SpectraMax M2 fluorescence microplate reader (Molecular Devices, San Jose, CA, USA) with excitation wavelength at 515 nm.

## 3. Results and Discussion

### 3.1. DNA Probe Design

Molecular detection of SARS-CoV-2 virus mainly relies on three regions that have highly conserved sequences. These sequences are (a) the RNA-dependent RNA polymerase gene (RdRP gene) in the open reading frame ORF1ab region, (b) the envelope protein gene (E gene), and (c) the nucleocapsid protein gene (N gene). Both the RdRP and E genes have been widely detected with a high sensitivity, whereas the N gene showed lower detection success with a much lower sensitivity [37,38]. This has attracted more attention to develop biosensors targeting the N gene sequence of SARS-CoV-2.

In this work, we chose the N gene as the model target to demonstrate the DSN-based COVID-19 detection in a proof of principle. The RNA target sequence is listed in Table 1, referenced from the amplification sequence of the N gene with the RT-PCR assay recommended by the National Institute for Viral Disease Control and Prevention (China). The RT-PCR assay depends on the specificity of primer binding. Once a mutation occurs in the primer-binding region, a false negative signal is given. To eliminate false negatives due to virus mutation at these specific regions, we designed two DNA probes for the virus RNA. One probe, N26, was for the primer-binding site of the recommended RT-PCR assay. Despite the point mutation, this approach can continue to amplify the fluorescence signal as long as about a 10 bp DNA/RNA perfect duplex can be formed. On the other hand, the other probe, N22, targets next to this site for a compensation to boost up the fluorescent signal. Therefore, this approach with two DNA probes could be used to pick up the mutated viruses. 

Both of the probes, N22 and N26, with a similar melting temperature (T_m_) of about 71 °C, were labeled with a Cy3 fluorescence reporter and a BHQ1 quencher. Cy3, which emits greenish yellow fluorescence, is one of the most popular cyanine dyes and is commonly used to label nucleic acids. BHQ1 has strong absorption range of 480–580 nm, which provides excellent quenching to the Cy3 fluorophores. To verify that the separation of the quencher, BHQ1, from the fluorophore, Cy3, can generate enough fluorescence signal for detection, we designed another two control probes, N22C and N26C, without the label of BHQ1, for a comparison. 

**Figure 1:**
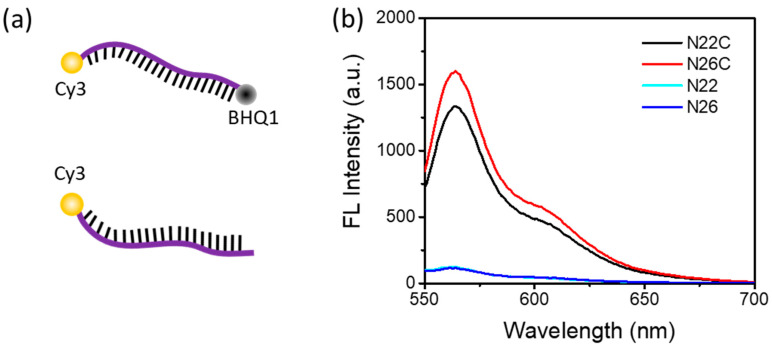
DNA probes and their fluorescence emission spectra (**a**) DNA probe dual labeled with a fluorophore and a quencher, or single labeled with a fluorophore. (**b**) Fluorescence emission of Cy3-labeled fluorescence probes in the presence or absence of the quencher BHQ1. The concentration of each probe used in these experiments was 1 μM.

Figure 1a shows the strands of DNA dual labeled with a fluorophore and a quencher, or single labeled with a fluorophore. As shown in Figure 1b, the fluorescence emission spectra show a maximum fluorescence emission peak at 562 nm with an excitation at 515 nm for the Cy3-labeled probes. With the fluorescence quencher BHQ1 close to Cy3, the fluorescence emission of Cy3 was significantly depressed. It indicated that when the DNA probe was cut into pieces so that Cy3 and BHQ1 were separated from each other, Cy3 fluorescence would be turned on to be detected. Therefore, these Cy3-labeled DNA probes were suitable for a DSN-based signal amplification method. 

It is worth noting that due to the different binding regions, these two probes could simultaneously hybridize to the target RNA and double the fluorescence intensity. Therefore, in the following experiments, both DNA probes were used in the assay to maximize the fluorescent signal. 

### 3.2. Optimization of the Incubation Temperature and Incubation Time

For the best detection performance, we firstly optimized the incubation temperature and incubation time considering that the enzyme efficiency varied with the temperature. In these experiments, 0.5 U DSN, 1 μM probe N22, 1 μM probe N26, and 100 nM target RNA were used in the assay. 

**Figure 2:**
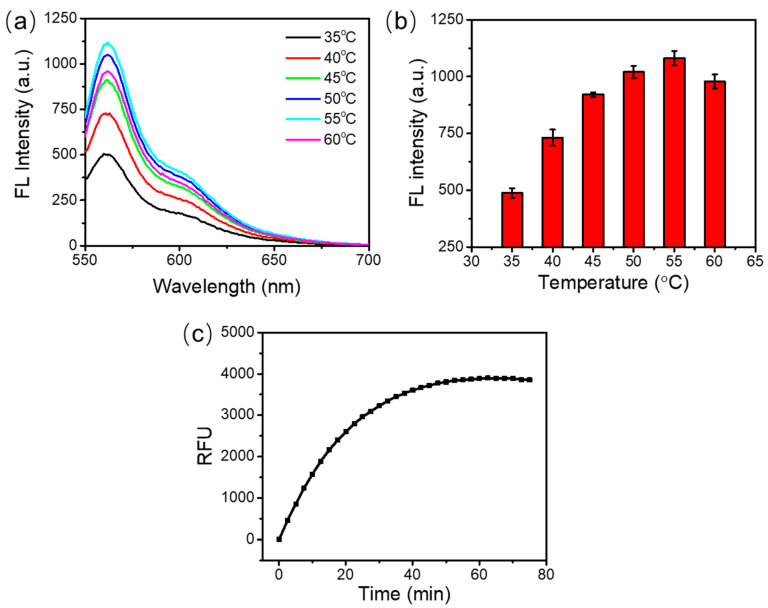
Optimization of the temperature and time for the duplex-specific nuclease (DSN)-based signal amplification. (**a**) Fluorescence emission spectra under the different reaction temperatures. (**b**) Peak values of the fluorescence intensity under the different incubation temperatures. (**c**) Real-time relative fluorescence units (RFU).

As shown in Figure 2a, the fluorescence emission spectra had similar shapes at various incubation temperatures from 35 °C to 60 °C. The maximum fluorescence emission signal was obtained at 55 °C (Figure 2b). A higher or lower temperature yielded lower fluorescence emission signals. This can be attributed to the inefficient hybridization between the DNA probes and the target RNA at a high temperature, or the reduced activity of DSN enzyme at a low temperature. Therefore, 55 °C was selected as the optimal reaction temperature. 

To optimize the incubation time, the assay was incubated at 55 °C in a thermal cycler which could record the fluorescence signal in real time. The fluorescence signal collection was achieved using the discrete excitation and detection wavelength ranges (excitation, 515–535 nm; detection, 560–580 nm). As shown in Figure 2c, the fluorescence signal increased with the extension of the incubation time from 0 to 60 min. The fluorescence saturated at around 60 min. After that, no obvious change of fluorescence signal was observed. This may be because all the DNA probes were used up after 60 min. The fluorescence reached 90% of the saturated signal at around 30 min. So, the reaction time can be set at 30~60 min. In our following studies, the detection of target RNA was carried out at 55 °C for 60 min.

### 3.3. Optimization of the Amount of DSN Enzyme

The amount of DSN in an RNA detection assay needs to be optimized given the fact that less DSN has lower enzyme activity and more DSN could generate nonspecific junk. In these optimization experiments, 1 μM probe N22, 1 μM probe N26, and 100 nM target RNA were used in the assays. The reaction was carried out at 55 °C for 60 min with 0.1 U, 0.25 U, or 0.5 U of DSN. A control reaction was run in parallel without any DSN included. 

**Figure 3:**
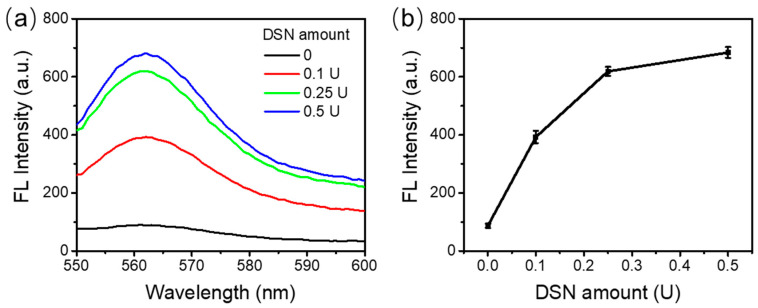
DSN enzyme activity. (**a**) Fluorescence emission spectra with various amounts of DSN enzyme. (**b**) The peak fluorescence values with different amounts of DSN enzyme.

As shown in Figure 3a, no fluorescence was observed for the control sample in the absence of DSN. The fluorescence recorded was the background fluorescence from the experimental setup. With an increasing amount of DSN, stronger fluorescence was observed. Figure 3b shows the values of peak fluorescence intensities. As can be seen, from 0 to 0.25 U DSN, a dramatic increase in the fluorescence emission spectrum was observed. However, the fluorescence increase from 0.25 U to 0.5 U DSN was not so significant, although it still showed an increment. We expected there would be no further improvement with more enzyme added. Considering the cost of the assay, 0.5 U DSN enzyme was used in the following experiments.

### 3.4. Enhancement of the Detection Sensitivity and Accuracy

Hypothetically, using multiple DNA probes, which can hybridize to different regions of SARS-CoV-2 RNA, could give a higher fluorescent signal, therefore improving the detection sensitivity and accuracy of the DSN-based signal amplification method. To verify this hypothesis, we investigated multiple probes in our assay and compared them with the single probe utilized. In these experiments, 0.5 U DSN, 100 nM target RNA, and two DNA probes (1 μM N22 and 1 μM N26) were added in the reaction mixture and incubated at 55 °C for 60 min. 

**Figure 4:**
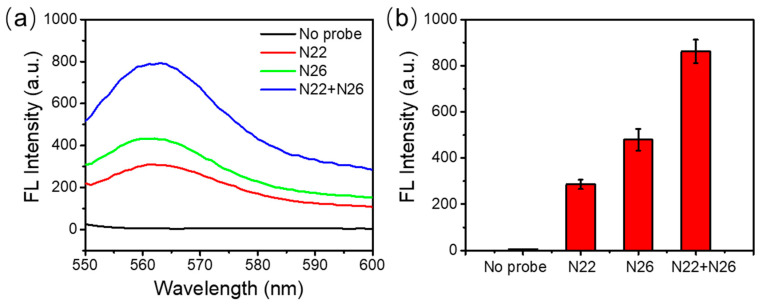
Multiple probe incorporation. (**a**) Fluorescence emission spectra with different probes. (**b**) The peak fluorescence values with different DNA probes.

As showed in Figure 4a,b, either N22 or N26 alone can give a clear fluorescence signal when the target RNA is presented. N26 has a slightly stronger fluorescence signal than N22. This may be due to the length of the two probes. N26 had 26 nucleotides while N22 had 22 nucleotides in total. With similar melting temperatures, hybridization of N26 to target RNA could offer more sites for DSN to recognize and to cleave for signal amplification. The combination of both of the probes in the same assay clearly gave significantly stronger fluorescence, as expected. Therefore, it was quite feasible to improve the detection sensitivity and accuracy by using multiple specific DNA probes. 

One thing that needs to be mentioned is that DNase I, a DNA-specific endonuclease that degrades DNA molecules for RNA analysis, may interfere with this assay by cutting the probes and releasing fluorescence. Therefore, DNase I or any other enzymes for DNA degradation should be avoided with this assay. 

### 3.5. Detection of SARS-CoV-2 RNA

Under the optimized conditions, we investigated the sensitivity of a DSN-based amplification method for SARS-CoV-2 detection. In these experiments, 1 μM probe N22, 1 μM probe N26, and 0.5 U DSN were used in the reaction while the target RNA concentration ranged from 500 pM to 500 nM. The reaction was incubated at 55 °C for 60 min before fluorescence recording. 

**Figure 5:**
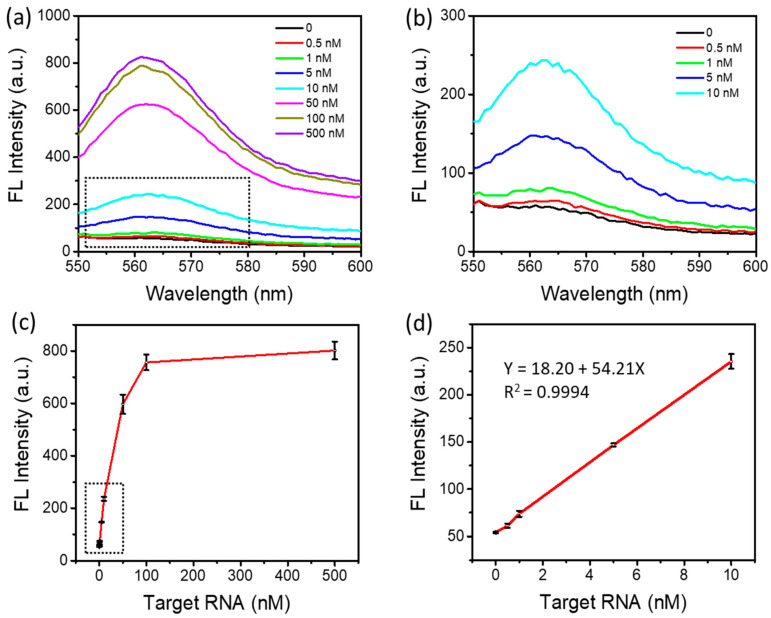
SARS-CoV-2 RNA detection. (**a**) Fluorescence emission spectra with different target RNA concentrations. (**b**) An enlarged view of the black dotted box in (**a**) representing the fluorescence responses to target RNA at low concentrations. (**c**) Fluorescence intensity depending on the concentrations of target RNA. (**d**) Linear relationship between fluorescence intensity and the concentration of target RNA, an enlarged view of the black dotted box in (**c**).

As shown in Figure 5a,b, a fluorescence intensity increase was observed when increasing the target RNA concentrations from 500 pM to 500 nM. The lowest detectable target RNA concentration was 500 pM. Figure 5c illustrates the changes in the peak fluorescence intensity depending on the target RNA concentrations. About 15-fold fluorescence enhancement was observed at the concentration of 100 nM above the background signal. As shown in Figure 5d, the fluorescence intensity was linearly dependent on the target RNA concentration in the range of 500 pM to 10 nM, with an R^2^ of 0.9994. The limit of detection can be down to approximately 500 pM, comparable to those of miRNA detection methods reported previously, as shown in Table 2. It is worth noting that such a relatively high sensitivity was achieved within 60 min in a one-step and simple detection method.

### 3.6. Selectivity of SARS-CoV-2 RNA Detection

**Figure 6:**
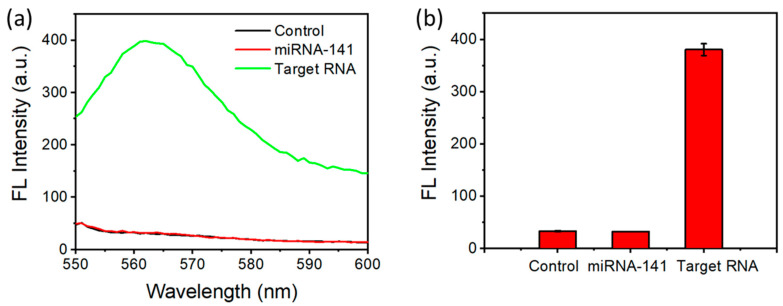
Selectivity of detection. (**a**) Fluorescence emission spectra with different RNA (100 nM). (**b**) The peak fluorescence values with different RNA.

To evaluate the selectivity of this approach where the signal only comes up when SARS-CoV-2 RNA is present, we conducted the experiments with miRNA-141 (an RNA sequence with 22 nucleotides) as a control group. No fluorescence signal was generated in the control group (Figure 6a,b), indicating the signal was specific to SARS-CoV-2 RNA. This was due to the mismatch between the probe and the RNA target, which did not allow the formation of the DNA/RNA heteroduplex to be cut by DSN. The selectivity was achieved by designing the sequence of the probe to be specific to SARS-CoV-2. 

## 4. Conclusions 

The gold standard method for SARS-CoV-2 nucleotide detection to date is based on real-time RT-PCR. This method needs an additional reverse transcription process and requires specific primers and probes, making the detection process and assay optimization complicated. Besides, changes in the viral genome due to nucleotide insertion, deletion, or recombination and interchange among viruses could easily cause false negative results. In this research, we develop a rapid, simple, and accurate direct RNA detection method with DSN-based signal amplification for SARS-CoV-2 detection. The detection method only needs a one-step reaction for 60 min at 55 °C. The results show that using multiple DNA probes could improve the detection sensitivity and accuracy. The limit of detection can be down to about 500 pM under the optimized conditions. The results also indicate that the more specific probes are in this detection method, the more sensitive and accurate the SARS-CoV-2 RNA detection can be. The method proposed here provides a new option for virus nucleotide detection in pandemic outbreaks.

## Figures and Tables

**Table 1:** The sequences of RNA and DNA oligonucleotides.

Name	Sequence (5′-3′)	Melting Temperatures (°C)
Target RNA	GUAGGGGAACUUCUCCUGCUAGAAUGGCU- GGCAAUGGCGGUGAUGCUGCUCUUGCUU	
N22	Cy3-CAAGAGCAGCATCACCGCCATT-BHQ1	70.8
N26	Cy3-AGCCATTCTAGCAGGAGAAGTTCCCC-BHQ1	71.9
N22C	Cy3-CAAGAGCAGCATCACCGCCATT	70.8
N26C	Cy3-AGCCATTCTAGCAGGAGAAGTTCCCC	71.9
miRNA-141	UAACACUGUCUGGUAAAGAUGG	

**Table 2:** Comparison with previously reported RNA detection methods.

Analyte	Linear Range	LOD	References
miRNA-21 (Fluorometric)	100 pM–30 nM	54 pM	[39]
miRNA-141 (Colorimetric)	20 pM–10 nM	20 pM	[40]
miRNA-29a-3p (MARS)	1 nM–70 nM	1 nM	[41]
Norovirus RNA (Microfluidic)	100 pM–3.5 nM	100 pM	[42]
SARS-CoV-2 RNA (RT-LAMP)		100 copies/reaction	[43]
SARS-CoV-2 RNA (RT-PCR)		0.15–100 copies/μL	[16,44]
SARS-CoV-2 RNA	500 pM–10 nM	500 pM	This work
